# Preclinical Large Animal *In-Vivo* Experiments for Surgically Implanted Atrioventricular Valve: Reappraisal and Systematic Review

**DOI:** 10.2174/1573403X18666220617115216

**Published:** 2023-01-01

**Authors:** Faizus Sazzad, Ramanathan Kollengode, Chan Li Xuan Beverly, Tan Ying Kiat, Geetha Ganesh, Theo Kofidis

**Affiliations:** 1 Department of Surgery, Yong Loo Lin School of Medicine, National University of Singapore, Kent Ridge, Singapore;; 2 Department of Cardiac, Thoracic and Vascular Surgery, National University Heart Center, Kent Ridge, Singapore

**Keywords:** Bioprosthesis, atrioventricular valve, mitral bioprosthesis, tricuspid, hydrodynamic, large animal, *in vivo*, pre-clinical

## Abstract

**Background:**

The development of atrioventricular bioprosthesis has witnessed an increasing drive toward clinical translation over the last few decades. A significant challenge in the clinical translation of an atrioventricular bioprosthesis from bench to bedside is the appropriate choice of a large animal model to test the safety and effectiveness of the device.

**Methods:**

We conducted a systematic review of pre-clinical in vivo studies that would enable us to synthesize a recommended framework. PRISMA (Preferred Reporting Items for Systematic Reviews and MetaAnalyses) guidelines were followed to identify and extract relevant articles.

**Results:**

Sheep was the most common choice of animal, with nine out of the 12 included studies being conducted on sheep. There were acute and chronic studies based on our search criteria. An average of ~20 and 5 animals were used for chronic and acute studies. One out of three acute studies and eight out of nine chronic studies were on stented heart valve bioprosthesis. All analyses were conducted on the implantation of atrioventricular valves with trileaflet, except for one chronic study on unileaflet valves and one chronic and acute study on bileaflet valves.

**Conclusion:**

Understanding the variance in past pre-clinical study designs may increase the appropriate utilization of large animal models. This synthesized evidence provides a pre-clinical *in vivo* studies framework for future research on an atrioventricular bioprosthesis.

## 1 INTRODUCTION

Pre-clinical studies are typically conducted to evaluate the feasibility of interventions before clinical studies are carried out. It includes *in vitro* biochemical studies and *in vivo* trials, including subcutaneous implantation in small animals and circulatory implants in large animals. In the context of atrioventricular valve design, pre-clinical *in vivo* trials evaluate the functional assessment, biocompatibility, immune response, imaging modalities, durability, and other pathological features of new atrioventricular valves prosthesis [1]. This is typically conducted *via* the implantation of the atrioventricular valve prosthesis in large animals ranging from sheep to monkeys. Pre-clinical *in vivo* studies hence play an essential role as a checkpoint to evaluate new mitral and tricuspid valve prostheses before human trials can be considered.

Depending on the study's objectives, pre-clinical studies can be acute (non-survival) or chronic (Survival experiments that examine the long-term impact on animals with a prosthetic valve). Acute studies usually only seek to evaluate the biocompatibility of new atrioventricular valve prostheses, and animals implanted with the valves are euthanized after a short duration. On the other hand, chronic studies assessing the rest of the parameters mentioned above, and hence animals implanted with a new atrioventricular valve prosthesis, are usually monitored for a more extended period (four weeks to more than 12 months). Despite the importance of pre-clinical studies in the atrioventricular valve design, there is significant variance in different pre-clinical study designs, outcomes reported, and animal models used. There are few recommended guidelines for pre-clinical study design on essential aspects of the study [2, 3]. These include the number of animals used, duration of follow-up, and the number of autopsied animals. Studies report hemodynamic data, survival, calcification, and thrombosis in different ways for outcomes reported. The type and species of animal used also vary greatly; while sheep is usually the most common, those studies also differ in the species and age. Over the years, lab research and comparisons focused on different pre-clinical animal models (such as dog, pig, calf, and sheep) used for cardiovascular implants, yet no standard model has been accepted.

The guidance provided by the US-FDA (United States Food and Drug Administration) in selecting appropriate animal models for cardiovascular devices is also not directed to a specific large animal. In addition, the published articles comparing swine, canine and ovine models, weighing each model’s advantages and disadvantages, are also not conclusive [4-6]. Therefore, pre-clinical study designs rely on the researchers’ discretion rather than a universal guideline. It is worth noting that different countries have different agencies regulating the use of animals in experimental studies. However, those agencies provide general broad-based principles not unique to atrioventricular valve prostheses. Hence, we conducted a systematic review of pre-clinical *in vivo* studies to synthesize a recommended framework that future researchers could use to develop an atrioventricular valve bioprosthesis.

## 2 METHODS

### 2.1 Data Sources, Search Strategy, and Study Selection

We conducted the systematic review electronically following the PRISMA (Preferred Reporting Items for Systematic Reviews and MetaAnalyses) guidelines [7]. Three reviewers searched Pubmed, Embase, and Web of Science for pre-clinical trials that evaluated any new atrioventricular valve bioprosthesis surgically implanted in large animals from inception to December 31^st^, 2021. The following search terms were employed to source eligible studies in repetitive and exhaustive combinations using ‘Medical Subject Headings’ (MeSH) headings: bioprosthesis, mitral valve, tricuspid valve, hydrodynamic, durability, and feasibility studies. There was no language restriction, but only papers written in English were included.

### 2.2 Enrollment Criteria

To be included, all studies had to evaluate mitral and tricuspid valve bioprosthesis in acute or chronic pre-clinical trials. Studies that reported concomitant other valve substitutes were included; however, only atrioventricular valve data were extracted. Only relevant studies that were related to the search terms were included. Studies where human patients were evaluated or mechanical valves were evaluated instead were excluded. Studies that used xenografts or implemented bioprosthesis *via* transcatheter atrioventricular valve replacement were also excluded. Studies that lacked details on the design of the pre-clinical trials, such as animal characteristics, type of prosthesis, and kind of clinical trials, were excluded. Retrospective studies, meta-analyses, systematic reviews, descriptive papers, case reports and series, ideas, editorials, and perspectives were excluded as their design was not pre-clinical large animal experimental trials. Studies that did not have an available English translation were also excluded.

### 2.3 Study Selection

Details of the flow of study identification are depicted in a Prisma flow diagram in Fig. (**1**). Database searches yielded a total of 564 citations after removing 558 duplicate sources. Five hundred forty-one irrelevant citations were excluded based on abstract and title evaluation. After further assessment of the full text of the remaining citations, 11 were excluded due to not meeting the selection criteria requirements. Thus, our analysis finally identified 12 eligible studies [8-20] comprising 199 animals.

### 2.4 Data Abstraction and Outcome

Three authors independently abstracted details of study design (type of valve used, number of animals, kind of pre-clinical trials, follow-up duration), characteristics of animals (species, gender, age, mean weight), size of prosthesis used, and outcomes of interest (hemodynamic performance, calcification, gross and histological findings). Characteristics of all included studies are summarized in Table **1**. Data synthesis was done utilizing the Review Manager software (RevMan 5.4).

The quality of the included studies was assessed by GRADE, as given in Table **2**. As illustrated in chapter 11 of the Cochrane handbook of reviews [21], GradePro evaluated the quality of evidence in the included studies. Authors assessed the articles for their risk of bias and quality of evidence by using Revmen 5.4. The risk of bias in each study was evaluated according to guidelines in chapter 8 of the Cochrane handbook of reviews [21]. We assessed each study based on its risk of bias, inconsistency, imprecision, indirectness, and publication bias per the Grade approach. The risk of bias and indirectness was judged as not serious for all studies as their study design was appropriate for pre-clinical trials. Inconsistency and imprecision were deemed not serious, even though each pre-clinical research had a different design and method of reporting since there was no standardized way of reporting outcomes for pre-clinical trials. Publication bias was undetected as each study reported on shortcomings of their valve implantation.

## 3 RESULTS

This systematic review included all studies that reported pre-clinical large animal trials, reporting data on new bioprosthetic atrioventricular valve replacement in three acute and nine chronic studies out of the 12 included studies.

### 3.1 Characteristics of the Included Studies

All the included manuscripts were single-center studies, mainly conducted in the United States and European countries, with one study from South Africa (Table **1**). Sheep was the most common choice of animal, with nine out of the 12 included studies being conducted on sheep. Most reported animals were three months to five years of age; most studies were not selective for animal sex. The average weight of the included study animals varied according to animal species, where 30-70 kg sheep were the most familiar choice. The follow-up duration of chronic studies ranged from three months to a year. An average of ~20 and 5 animals were used for chronic and acute studies.

#### 3.1.1 Animal Species and Rationale of Selection

Researchers have utilized different animal models for the atrioventricular valve *in vivo* evaluation. The rationale for animal characteristics is included in Table **2**. Sheep have been associated with most of the studies, but the use of different sheep species was variable and largely dependent on the availability and geographical distribution. Use of Juvenile sheep was found in most cases [10, 11, 17, 20], while Collatusso *et al*. used Texel juvenile sheep [8] and Navia *et al*. used Dorset sheep species [15]. Paravicini *et al*. and Thiene *et al.* [16, 19] have reported using adult sheep with unknown species.

#### 3.1.2 Outcomes

All outcomes of interest (hemodynamic performance, calcification, gross and histological findings) were extracted and presented in Table **3**. Keeling *et al*. [14], Navia *et al*. [15], and Spyt *et al*. [18] have tested stentless mitral prosthesis, whereas other authors used a stented bioprosthesis. Most of the bioprosthesis were sterilized using different concentrations of glutaraldehyde, except Flameng *et al*. [10], who used 100% ethylene oxide. The early termination of studies was as high as 50% for Collatusso *et al*. [8] and Thiene *et al*. [19] studies.

## 4 DISCUSSION

During the past several years, remarkable achievements have been made in the pre-clinical evaluation of valve prostheses using experimental animal models, *i.e.*, pig, dog, or sheep [22]. For research that focuses on clinical translation, it is vital that the preliminary results obtained from the study on the small rodent animals can be confirmed by performing the same research on a larger animal model which has a higher resemblance to the humans with a more significant percentage of the genetic conservation [23]. The choice of animal models for research purposes is critical as well. Therefore, a conceptual flow diagram has been depicted in Fig. (**2**), describing the decision-making steps before proceeding with a large animal pre-clinical experiment. The flow chart has been compiled following General Considerations for Animal Studies for Medical Devices provided by US FDA [24].

The most evident and demonstrative reason behind the choice is the difference in gross anatomy between humans and animals and between animals of various species. The gross anatomical difference implies that the structures with the same anatomy can have other functions. Thus, they can be subjected to different biomedical strains as well. Since the primates, which closely resembles humans, cannot be used as an animal model for research purposes because of their high cost, the most frequently used animal species are the canines and swine. This resulted in a vigorous debate on the animal species with a higher resemblance to humans. However, their salient anatomical and functional differences should also be considered before using pigs and dogs. For example, there are differences between the coronary circulation of pigs and dogs. In pigs, the coronary circulation does not contain any collateral connection between the vascular branches, whereas, for dogs, it can be extensive [25]. So, generally, the young human heart resembles a pig's heart, and as the heart gets older with ischemic heart disease, it will resemble a dog's heart.

To answer all research questions, ideally, studies should not depend on a single animal model as an ideal large animal model of the human cardiovascular system does not exist. However, the difference between humans and large experimental animals decreases when animal models' body or heart weight approaches humans [26]. There are various advantages of conducting *in vivo* studies with the help of large mammalian hearts, namely, clinically relevant, physiological, perform survival experiments, cardiac functions and responses assessment in large animals, and the best for the testing a new device because of the conserved structural continuity [25].

*Ovis aries* is a docile, domestic sheep that provides an extensive opportunity in research by acting as an experimental animal model [27] because of their unique acceptance as a research animal [28]. Sheep require relatively inexpensive housing and feeding; they are obedient, making them easy to handle. The average size of the sheep is about 50-90Kg with much resemblance to humans compared to other large animals. Therefore, it will help perform cardiac device development testing for various anatomical and structural locations. The body shape and size of the sheep are also compatible for imaging studies like transthoracic echocardiography, CT (computerized tomography) scan, or MRI (magnetic resonance imaging), which were designed for humans. Concurrently, it also allows for performing various surgical procedures and medical devices, such as tissue-engineered heart valve prostheses, bioengineered prostheses, or implantable devices. On the contrary, based on the aim of the study, which is being conducted on the sheep, the potential benefits of using them have to compensate for some technical constraints. For example, currently available reagents should improve for sheep genome studies; the cardiovascular cannulation technique for sheep largely varies from other animals. A cervical access and neck cannulation for establishing cardiopulmonary bypass requires additional surgical skills. However, the preoperative and perioperative use of medications and the surgical equipment in sheep surgery are similar to larger animals like a horse. Therefore, the significant investments required in the extensive and specialized handling equipment and the surgical tables can be avoided using sheep as the animal model. Concurrently, the sourcing of sheep is comparatively easy and less costly. They are also considered research animal models that are socially acceptable and have fewer ethical issues raised relative to companion animals [29]. Since sheep are large animal models that more resemble the human heart, they are approved to be served as an excellent pre-clinical animal model for cardiovascular research purposes [30]. From the functional perspective, the myocardial contractility and relaxation mechanism in sheep are similar to the myocardium in humans. Cardiodynamics, *i.e.*, systolic and diastolic pressure in sheep (~90 – 115 mmHg), is identical to that in humans (90 – 120 mmHg), and so is the heart rate in the sheep (60-120 bpm). There is wide use of the sheep model for cardiovascular applications, mainly used to test the heart valves since the valve anatomy of sheep and humans are similar and since the size of the sheep permits access to the aortic and mitral valve and pulmonary valve [31]. Moreover, the ovine model is considered the best animal model for valve replacement survival studies to satisfy the US FDA [24] and CE mark requirements. The cardiovascular anatomy and physiology in ovine models are regarded as an accepted *in vivo* model that reliably mimics the cardiac anatomy and physiology of the human for the safety and performance evaluation of a cardiac implant.

Swine are the most widely used animal model for pre-clinical large animal experiments because of their similar organ size, coronary anatomy, and cardiac structural compartments. Compared to other animal models, porcine models can be identical to humans in terms of cardiovascular and gastrointestinal anatomy and physiology. Pigs also acquire a fast substantial litter size, have an early maturity, and breed year-round, hence considered the most suitable animal model for biomedical research programs [32]. Due to these structural and developmental similarities, where younger pigs have a body and size similar to the adult human, they are widely used as an animal model in cardiovascular device development, organ transplantation, and other surgical procedures [33]. They are also used as a pre-clinical model in drug discovery [33] and many other genetic models of human diseases [32, 33]. Pigs are also gaining attention for the study of tissue engineering (*i.e.*, tissue-engineered heart valves) and also in regenerative medicine (*i.e.*, stem cell therapies), applications (*i.e.*, transcatheter heart valve implantation), and the field of biomechanical studies. The exponential rise of publications focused on these swine experiments during the last 30 years serves as a shred of good evidence [33]. For the past few decades, pigs have been widely used to teach and research animal models for surgeries. Since the 1990s, pigs have been considered the default model for surgical teaching, substituting dogs. Furthermore, their omnipresence and educational use enabled them to be widely adopted in various models for organ transplantation, such as heart, lungs, liver, and kidney [33]. Moreover, in the case of cardiac bioprosthesis manufacturing, pigs have been used as xenograft donors, where initially, porcine valves were transplanted into non-human primates, which has been later translated into wide-scale human implantation. As a result, several research groups focused on humanizing the pigs by conducting several studies for eliminating the major xeno-antigen(s) in the porcine genome, which was recognized by the natural human antibodies. Furthermore, gene deletion or pharmacotherapy has also addressed PERV (porcine endogenous retrovirus) related zoonosis to reduce the associated risks [34]. All these are substantial evidence for using the swine model in multiple large animal experimental research. Despite having a lot of similarities between swine and human hearts, there are some limitations to swine models. The porcine heart is relatively hypersensitive and may frequently lead to refractory arrhythmias due to anesthesia and surgical manipulation, and these are refractory to multiple defibrillation efforts on many occasions.

Domesticated dogs are variable models for various biomedical research, namely, aging and Alzheimer's Disease, naturally occurring genetic diversity, NHL (Non-Hodgkin's Lymphoma), and other cancer (*i.e.*, Osteosarcoma), and limited cardiovascular interventions. Dogs have been commonly used for respiratory system studies, as canine lung mechanics, ventilation, cough reflex, and central neuronal control mechanisms are similar to humans. Asthma and COPD (Chronic Obstructive Pulmonary Disease) are most extensively studied in the canine model as these disease patterns closely resemble human chronic bronchitis [18]. The unique advantage of the canine model over other commonly used experimental animals is the shorter duration for disease development and progression. There are similarities and differences between the canine and the human cardiovascular system. Critical data related to ischemia cascade and myocardial infarction in the canine model is significantly different from humans. Coronary occlusion may not ultimately lead to necrosis, LV remodeling, and heart failure in canines due to the extensive presence of collateral coronary circulation [25]. As a result, the canine heart is used as a large animal model in myocardial ischemia research keeping the significant drawback of variable reperfusion, resulting in a varying degree of post-MI remodeling. However, the use of canine models for cardiovascular device testing or heart valve implantations is not widely practiced at the moment.

Some researchers prefer female pigs over male pigs, though both can be used. The reason for the preference for female pigs is that urinary catheterization is more straightforward in females because the female urethra is shorter and more straightforward than the male urethra, which is corkscrew-shaped and has an angulated path to the urinary bladder. In addition, urine output is an excellent indirect peri-operative measure of circulatory status and response to medications used to treat abnormal hemodynamic, acid-base, and electrolyte balance states, so monitoring it is an essential part of cardiopulmonary bypass. However, the research team should consider using male pigs if (a) female pigs are unavailable, causing a significant delay in the study, and (b) urinary catheterization of male pigs can be performed in a satisfactory and less traumatic manner to monitor urine output in the peri-operative period. Although not explain the reason, the studies conducted on calves by Daebitz *et al*. [9] included all female animals, similar to that by Sypt *et al*. [20], where all included sheep were female.

Age should be considered when selecting an experimental large animal model [35]. Animal trials are frequently conducted in juvenile or neonatal animals for practical and organizational reasons. The use of juvenile sheep by Flameng *et al*. [10], Fukamachi *et al*. [11], Shemin *et al*. [17], and Wheatley *et al*. [20] was significantly different from Paravicini *et al*. [16] and Thiene *et al*. [19]. While the former group was using growing sheep, the latter chose adults. It is worth noticing that selecting an adult model largely depends on the species’ growth. Some adult sheep grow in 35-50 kg by 3-4 years, depending on the species. However, differences in healing, recovery from surgery, and mineralization responses between juvenile and adult animals, on the other hand, can skew the results of such trials [36]. As a result, an optimal study design is mainly dependent on the following factors, namely, appropriate age of the animals, skeletally mature, ability to mimic human disease, and healing and recovery potentials. The animals used should be within comparable age levels to accurately reflect human age-related disease. The use of body weight (with ranges) is a critical demographic parameter for all animal studies to include in the experiment report. However, body weight and age are interdependent and directly correlate with the animal's life cycle, living, and species. In addition, animal acclimation, husbandry, animal care facility, and environmental factors significantly impact body weight, often through poorly understood interactions. Since weight may affect many other biological outcomes [37], it seems rational to include it in the animal description and age. However, all animals undergoing dietary manipulations and restrictions need to be regularly monitored for body weight loss (*e.g*., weighing animals three times a week). A proper record of animal weight should be kept, and animals that have lost 20% of their body weight must be euthanized or excluded from the study.

One of the most crucial phases of pre-clinical large animal experiments involving surgically implanted mitral valves usually involves juvenile sheep or pigs. In such model systems, hemodynamic performance for safety and effectiveness assessment of a mitral bioprosthesis can be assessed, including transvalvular gradient, effective orifice area, *in vivo* hemodynamics, ease of implantation, device description, injury to surrounding cardiac structures, and durability assessment (*i.e.*, fatigue analysis and life cycle testing). Mineralization of the bioprosthesis is a specific consideration that may determine the long-term use of a device in humans; it should be tested with caution in a sheep model. Sheep is a known highly mineralizing animal, hence, using sentient animals for research may require cautious evaluation.

The common mitral prosthesis is stented, even though few stentless mitral prostheses in development are also tested in pre-clinical large animal models [14, 15, 18]. On the other hand, Flameng *et al*. [10] used a 100% EO (ethylene oxide) to sterilize their prosthesis in a chronic sheep model that did not report calcification. The geographical distribution primarily determines the availability of an animal model. For example, Chacma baboons by Hassoulas *et al*. [12, 13] and calves by Daebritz *et al*. [9] are infrequent and limited. Another limitation we faced was the scarcity of experimental models available in the literature studying MVR (mitral valve replacement) procedures with a bioprosthesis. Therefore, further exploration and analysis need to be performed with a larger cohort to provide a piece of more substantial evidence for an optimal animal model for mitral valve replacement surgery.

## CONCLUSION

Animal models are still a valid method of pre-clinical testing, especially for the multiple regulatory authority, for the device development that cannot be replaced by the benchtop, *in-vitro*, *ex-vivo* or *in-silico* experiments. In conclusion, our review described the usefulness of large animals and the rationale for selecting an animal model for testing mitral bioprosthesis and shared a categorical decision-making algorithm for new device testing in an experimental large animal model. Although researchers prefer to opt for testing the device as a conveniently available animal model for mitral valve replacement, our review suggests that the justified use of animals in a stepwise ladder is the rational alternative. However, understanding the variance in past pre-clinical study designs may increase the appropriate utilization of large animal models for *in-vivo* testing of the mitral bioprosthesis, which is mutually beneficial for animals and respective researchers.

## Figures and Tables

**Fig. (1):**
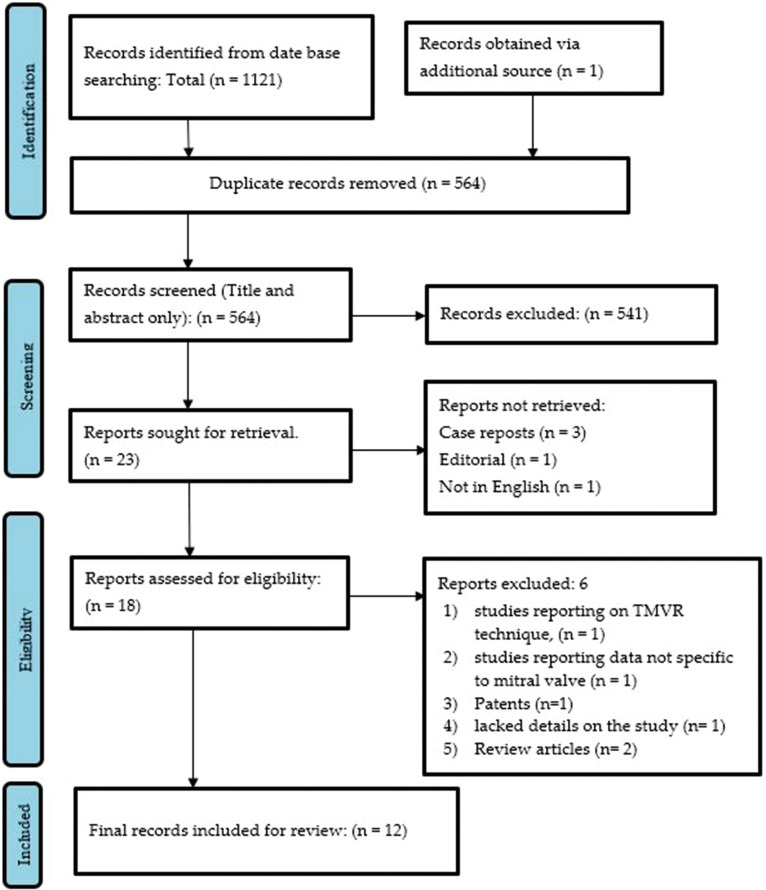
PRISMA chart illustrating our process of obtaining the 12 included articles.

**Fig. (2):**
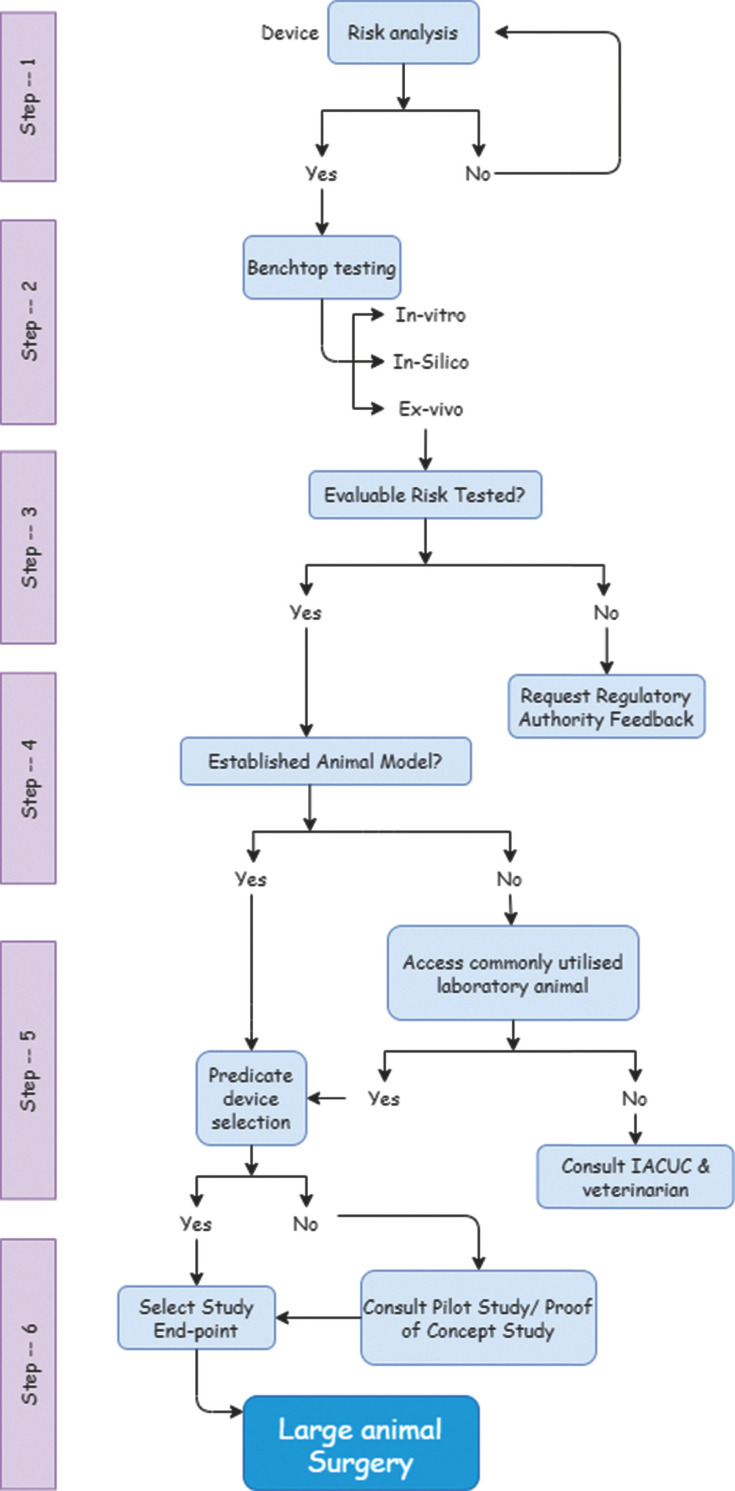
Decision-making algorithm for large animal surgery.

**Table 1:** Characteristics of the included *in-vivo* studies for mitral bioprosthesis pre-clinical evaluation.

**Author, Year**	**Type of Device**	**Study Type**	**Place**	**Number (n=199)**	**Animal**	**Age**	**Sex**	**Mean Weight (kg)**	**Follow up**
Collatusso 2018	dCell bovine pericardium valves	Chronic	Brazil	16	Sheep	5-6 months	-	35±3 [30-40]	6 months
Daebritz 2003	ADIAM biomechanical valve	Chronic	Germany	14	Calves	3–5 months	F	79±9 [60–97]	21.8±0.4 weeks
Flameng 2015	Perimount mitral valve	Chronic	Belgium	45	Sheep	<6 months	-	22- 38	8 months
Fukamachi 2007	CE bovine pericardial valves	Acute	USA	03	Sheep	-	-	67.7±21.1	NA
Hassoulas 1988	Microflow Pericardial Valve	Chronic	South Africa	37	Baboons	Young	-	21.9±5.7 [15-32]	12 months
Keeling 2007	Modified Toronto stentless valve	Acute	Florida, USA	02	Swine	-	-	100	NA
Navia 2007	Stentless pericardial mitral valve	Acute	Ohio, USA	10	Sheep	-	-	73±9	NA
Paravicini 2012	Mitral valves from tuna cornea	Chronic	Brazil	09	Sheep	3–4 years	-	35–50 kg	180 days
Shemin 1988	Unileaflet pericardial bioprosthesis	Chronic	New Jersey, USA	09	Sheep	3-5months	-	22-33	3-5 months
Spy 1988	A new three-leaflet pericardial bioprosthesis	Chronic	Glasgow, UK	18	10 sheep, 08 dogs	18-20 months (sheep)	F/Sheep	45-60 (sheep), 25-33 (dogs)	3 months
Thiene 1989	Pericarbon by Sorin	Chronic	Padua. Italy	22	Sheep	3-5 years	-		~469 days
Wheatley 1999	Polyurethane valve	Chronic	Glasgow, UK	14	Sheep	21 ± 4 [17-25 weeks]	-	-	6 months

UK= United Kingdom, USA=United States of America, NA=Not Applicable,

**Table 2:** Animal species, characteristics, the rationale of selection.

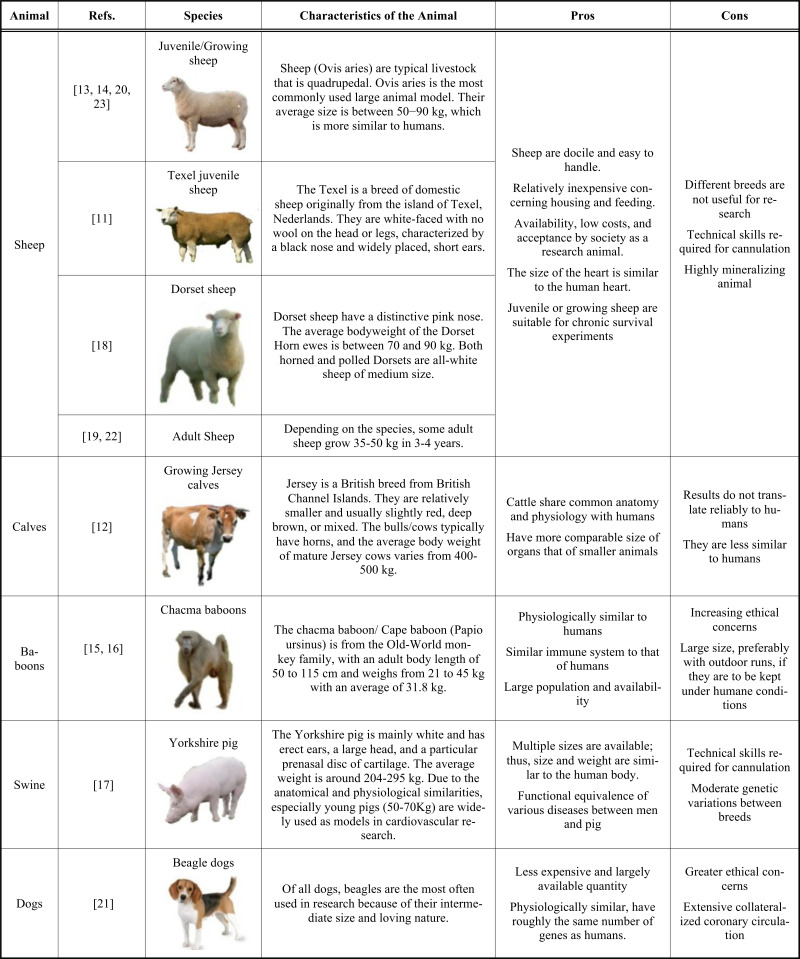

NB: Animal information and images were collected from the open-access internet. Available from https://en.wikipedia.org/wiki/Main_Page

**Table 3:** Characteristics of the included in-vivo studies for mitral bioprosthesis pre-clinical evaluation.

**Author, Year**	**Device**	**Cusp**	**Size of the New Prosthesis**	**ΔP (mmHg)**	**Number**	**Survival**	**D/T**	**Tx**	**Et**	**ST**	**C**	**Hx**
Collatusso [11]	S	3	23mm	10±2	16	8	8	-	-	0.5% glut	2	Macro
Daebritz [12]	S	2	29mm	8.4±5.3	14	14	0	1	-	-	6	Macro
Flameng 2015 [13]	S	3	25mm	3.9±1.6	45	31	14	-	1	100% EO	-	H&E, VK
Fukamachi [14]	S	3	25mm, 27mm	-	03	NA	-	-	-	-	-	Macro
Hassoulas [15,16]	S	3	21mm	9.73±4.93	37	33	4	-	0	0.2-0.4% glut	4	Nil
Keeling [17]	SL	3	-	-	02	NA	-	-	-	-	-	Macro
Navia [18]	SL	2	-	2.1±1.2	10	NA	-	-	-	0.65% glut	-	-
Paravicini [19]	S	3	23mm	-	09	9	0	-	-	0.3% glut	0	Macro
Shemin [20]	S	1	23mm	2.4±0.3	09	-	-	0	2	Chemically treated	4	H&E, VK
Spy [21]	SL	3	Sh: 25mm D: 21mm	Sh:2.51 [1.8-3.^8^] D:5.2 [5-5.^5^]	18	Sh:7 D:4	Sh:2 D:2	Sh:0 D:2	Sh:1 D:1	-	Sh:4 D:0	H&E, VK
Thiene [22]	S	3	25,27,29 mm	-	22	11	11	-	2	0.5% glut	4	H&E, VK
Wheatley [23]	S	3	24mm	3.0±0.95	14	6	8	-	-	-	-	H&E, VK, SR

**Abbreviations:** S=Stented; SL=Stentless; Tx= Throbmosis; D/T= Death/Termination; Et= Endocarditis; ST= Sterilization; C= Calcification; Sh= Sheep; NA= Not applicable; Glut= Gluteraldehyde; EO= Ethylene oxide; Hx= Histology; H&E= Hematoxylin and eosin; VK= Von Kossa; SR= Sirius Red; Macro= Macroscopic examination.

## Data Availability

Research data are available upon request for corresponding author [FS].

## LIST OF ABBREVIATIONS

PRISMA: Preferred Reporting Items for Systematic Reviews and MetaAnalyses
CT scan: computerized tomography scan
MRI: magnetic resonance imaging
US-FDA: United States Food and Drug Administration

## SUPPLEMENTARY MATERIAL

PRISMA checklist is available as supplementary material on the publisher’s website along with the published article.
